# Advances in the surgical treatment of esophageal cancer since 1965

**DOI:** 10.1002/ags3.12332

**Published:** 2020-04-14

**Authors:** Hisahiro Matsubara

**Affiliations:** ^1^ Department of Frontier Surgery Graduate School of Medicine Chiba University Chiba Japan

**Keywords:** development, esophageal cancer, history, surgical treatment

## Abstract

In Japan, the treatment of esophageal cancer has undergone significant development since the Japanese Society for Esophageal Diseases was established in 1965 by Doctors Komei Nakayama, Shigetsugu Katsura, and Ichiro Akakura. When the Society was established, surgery was the first‐line treatment for esophageal cancer. Since then, the Society has been led by three successive chairpersons—Doctors Katsura, Nakayama, and Satoh. Over this time, surgery‐related mortalities declined to 5%‐6% because of the rapid improvements in surgical technique. Beginning in 1980, the bilateral cervical lymph node dissection technique gained attention, and favorable long‐term outcomes were gradually reported. A nationwide questionnaire survey, conducted by the Society in 1990, showed that more favorable long‐term outcomes were achieved by following the three‐field lymph node dissection technique than by following the two‐field lymph node dissection technique. Since then, the three‐field lymph node dissection technique has been recognized and widely used as the standard surgical procedure for treating esophageal cancer. After clinical studies examined the utility of various pre‐ and postoperative adjunctive therapies in outcome improvements, preoperative chemotherapy was recognized as the standard treatment in the therapy guidelines. Additionally, less invasive surgical methods have been developed, including endoscopic and robot‐assisted surgeries, which are applied in general practice now. However, unresectable and recurrent esophageal cancers remain difficult to treat, and additional treatments should be developed.

## 1 INTRODUCTION

In 1932, a new era in the treatment of esophageal cancer dawned in Japan when Doctors Sadanobu Seo (Second Department of Surgery, Chiba University) and Tohru Ohsawa (Kyoto University) presented their assigned reports at the Annual Meeting of the Japan Surgical Society.^1^, ^2^ The establishment of the Japanese Society for Esophageal Diseases (predecessor of the current Japan Esophageal Society) was primarily initiated by Doctor Komei Nakayama in 1965. Following this establishment, the field of esophageal cancer treatment underwent important advancements. Moreover, due to the efforts of our predecessors, surgery was selected as the first‐line treatment for this disease; the significance of the three‐field lymph node dissection technique was recognized and has been widely used. Besides, other relevant developments happened to reduce the invasiveness of surgical treatment. This review describes these milestones in the surgical treatment of esophageal cancer and the advancements thereafter.

## 2 DAWN OF A NEW ERA

The world's first report of a successful thoracic esophageal cancer treatment was published by Torek in 1913.^3^ In that report, a patient underwent a two‐stage surgery and survived for an additional 13 years. Reconstruction was not performed, but an artificial esophagus, made from a rubber tube, was placed between the cervical esophageal fistula and the gastric fistula. In the same year, Fink attempted to perform antethoracic esophagogastrostomies, although the first successful case was reported 6 years later, in 1920, by Kirschner.^4^

In Japan, the reports published by Professors Sadanobu Seo (Second Department of Surgery, Chiba University School of Medicine) and Toru Osawa (Kyoto University) are considered to represent the dawn of a new era in esophageal cancer treatment. In 1929, Doctor Toru Osawa successfully treated a patient by conducting a total gastrectomy and an intrathoracic esophagojejunal anastomosis, via laparotomy and thoracotomy.^5^ In 1932, Professor Sadanobu Seo reported the case of a patient who was cured after undergoing an intrathoracic esophagogastrostomy immediately after esophageal cancer resection in the Journal of Japan Surgical Society. A special article published in the Journal of Japan Surgical Society in 1933 indicated that prior to February 1932, six patients with cervical esophageal cancers and 16 with thoracic and abdominal esophageal cancers underwent surgical resection at the Second Department of Surgery at the Chiba University School of Medicine, with mortality rates of 16.7% and 50.0%, respectively.^1^ Several reports of successful cases involving the resection of thoracic esophageal cancers were reported, including five cases by Professor Sadanobu Seo and two by Professor Eggers. The other cases included the previously described case reported by Torek and five reports of single cases, each authored by different individuals.^1^ While analyzing the studies published on the surgical outcomes of patients who underwent resections of thoracic or abdominal esophageal cancers before 1924, a mortality rate of 95.4% was observed. This seems like an event from another time, compared with the mortality rates achieved today with resections of esophageal cancers.^1^

Later, in the 1950s, various anastomotic methods for conducting esophageal cancer surgeries were reported, and various European and American authors reported improvements in the surgical outcomes for upper‐ and middle‐thoracic esophageal cancers, although the mortality rate remained at 20%‐50%.^6^ Professor Komei Nakayama (Second Department of Surgery, Chiba University School of Medicine) authored a special article published in 1951, reporting the treatment of 30 patients with a mortality rate of 16.7%.^6^ European and American authors reported that, mainly, intrathoracic anastomoses were performed, whereas the safety of treatments improved after antethoracic esophagogastrostomy was introduced.

Concurrent with these technical improvements, anesthesia also progressed. Beginning in 1950, the Second Department of Surgery at Chiba University School of Medicine started to use ether administered via endotracheal intubation as a general anesthetic during esophageal cancer surgeries. Prior to that, surgeries had been performed using a combination of lumbar spinal anesthesia and local anesthesia.

## 3 FIFTY YEARS OF PROGRESS SINCE THE JAPANESE SOCIETY FOR ESOPHAGEAL DISEASES WAS ESTABLISHED

After the publication of the assigned reports, the aforementioned advances occurred gradually and were recognized as major developments over the 50 years since the Japanese Society for Esophageal Diseases (predecessor of the current Japan Esophageal Society) was established in 1965 (Table 1). At the time of the establishment of the Society, it was led by Doctors Komei Nakayama, Shigehiro Katsura, and Ichiro Akakura, whose efforts led to surgery becoming the first‐line treatment for esophageal cancer.

**TABLE 1:** Major developments over the 50 y since the Japanese Society for Esophageal Diseases

Year	Month	Meeting number	Topic	Facilitator/Chairman	Venue	Event
1965	October	1st meeting	Postoperative complications of esophageal cancer/Esophageal reconstruction techniques in esophageal cancer	The late Doctor Ichiro AKAKURA	Tokushima	August 1965: The Japanese Society for Esophageal Diseases was founded
1966	October	2nd meeting	Nutritional management before and after esophageal cancer surgery/Proposed Clinical Classification for Carcinoma of Esophagus/Movie “Idiopathic esophageal dilatation”	The late Doctor Hiroshi SATO	Tokyo	
1967	October	3rd meeting	Esophageal reconstruction/Clinical Classification for Carcinoma of Esophagus	The late Doctor Shigeru HATANO	Nagoya	
1968	July	4th meeting	Interesting cases of esophageal cancers treated via preoperative irradiation/Discussion on the Clinical Classification for Carcinoma of Esophagus	The late Doctor Komei NAKAYAMA	Tokyo	
	October	5th meeting	Early detection of esophageal cancer/Study of fatal cases of esophageal cancer/Clinical Classification for Carcinoma of Esophagus	The late Doctor Takeo HAYASHIDA	Tokyo	April 1969: The first edition of the “Clinical Classification for Carcinoma of Esophagus” was published
1979	May	26th meeting	Preparation of a gastric tube for esophageal reconstruction: theory and practice/Esophageal cancer with metastasis or multiple primary cancers	The late Doctor Toshifumi IIZUKA	Tokyo	Year 1979: Establishment of the International Society for Diseases of Esophagus (ISDE)
	November	27th meeting	Cervical esophageal cancer/postoperative pulmonary complications	Doctor Teruo KAKEGAWA	Tokyo	
1980	May	28th meeting	Clinical course of esophageal cancers from a retrospective standpoint/Diagnosis, pathology, treatment, and prognosis of submucosal invasive cancers	The late Doctor Hikoo SHIRAKABE	Tokyo	November 1980: 1st Congress of the ISDE in Tokyo/Japan
	November	29th meeting	Treatment of esophageal varices/Composite resection including other vital organs in thoracic esophageal cancers	The late Doctor Mitsuo ENDO	Tokyo	
1981	May	30th meeting	Esophageal cancers; host and environment/Carcinoma developing in a reconstructed esophagus	Doctor Hiroshi AKIYAMA	Tokyo	
2002	June	56th meeting	Evaluation of radiation therapy and chemotherapy for esophageal cancers/Minimally invasive therapy for esophageal cancers/New diagnostic methods for esophageal cancers	The late Doctor Tetsuya TOGE	Hiroshima	December 2002: The “Guidelines for the Treatment of Esophageal Cancers” was published
2003	June	57th meeting	Establishment of the Japan Esophageal Society	Doctor Masayuki IMAMURA	Kyoto	January 2003: The Japan Esophageal Society was established
2004	June	58th meeting	Aiming for a state‐of‐the‐art diagnosis and treatment of esophageal diseases—Cross‐sectional verifications that go beyond specialties	Doctor Masaki KITAJIMA	Tokyo	
2005	June	59th meeting	Carrying on the tradition of wisdom	The late Doctor Teruo KOZU	Tokyo	
2006	June	60th meeting	Diagnosis and treatment of esophageal diseases from the perspective of basic sciences, internal medicine, radiaology and surgical specialties	Doctor Masahiko TSURUMARU	Tokyo	
2007	June	61st meeting	Aiming to integrate knowledge and practice: Consistency between diagnosis and treatment	Doctor Hiroyasu MAKUUCHI	Yokohama	April 2007: The tenth edition of the “Japanese Classification of Esophageal Cancer” was published. The “Guidelines for the Diagnosis and Treatment of Esophageal Cancer” was published
2008	June	62nd meeting	Accumulating knowledge about esophageal diseases	Doctor Kaiyo TAKUBO	Tokyo	September 2008: 11th Congress ISDE in Budapest/Hungary
2009	June	63rd meeting	Overcoming esophageal cancer via team medical care	Doctor Nobutoshi ANDO	Yokohama	
2010	August	64th meeting	Japan Esophageal Society Consensus Meeting 2010: Where are we now?	Doctor Hiromasa FUJITA	Kurume	September 2010: 12th Congress ISDE in Kagoshima/Japan
2011	September	65th meeting	Conducting a careful observation of tubes rather than asking for answers from Heaven: Patient‐centered multidisciplinary care	Doctor Shogo YAMADA	Sendai	
2012	June	66th meeting	The past and the future of esophageal diseases	Doctor Hiroyuki KUWANO	Karuizawa	April 2012: The “Guidelines for the Diagnosis and Treatment of Esophageal Cancer” was published
2013	June	67th meeting	Challenges for the future	Doctor Harushi OSUGI	Osaka	
2014	July	68th meeting	Determining the limitations of diagnosis and treatment	Doctor Kumiko MONMA	Tokyo	
2015	July	69th meeting	Challenges and validation	Doctor Soji OZAWA	Yokohama	October 2015: The eleventh edition of the “Japanese Classification of Esophageal Cancer” was published
2016	July	70th meeting	Simplified study of the esophagus and recommendations therein: Learning esophagology in a fun and instructive manner	Doctor Harushi UDAGAWA	Tokyo	
2017	June	71st meeting	The boldness to forge ahead, and the calm courage to retreat	Doctor Tsuneo OYAMA	Karuizawa	June 2017: The “Guidelines for the Diagnosis and Treatment of Carcinoma of the Esophagus 2017” was published
2018	June	72nd meeting	ShuHaRi ‐ Keep, Break, Open up ‐	Doctor Hiroyuki KATO	Utsunomiya	
2019	June	73rd meeting	“Seed and Soil” in Esophagology	Doctor Yasushi TOH	Fukuoka	

In addition to the aforementioned antethoracic esophagogastrostomy, Doctor Nakayama also invented a safer three‐stage procedure in which a gastrostomy is created to facilitate improving patient nutrition during the first stage. Since this method was introduced, the importance of preoperative nutritional management has been considered.^7^ At the same time, preoperative radiotherapy is performed to inhibit the progression of cancer. The second stage of the surgery involves esophageal resection and observation of the clinical courses of the cervical esophagostomy and gastrostomy over several months to 1 year. Reconstructive surgery, using antethoracic esophagogastrostomy, is performed during the last stage. A report published in 1964, in the Japanese Journal of Clinical and Experimental Medicine, showed that the surgery‐related mortality was 7.4%.^6^ This was an epoch‐making outcome and in addition to safety improvements, the 5‐year survival rates were previously single‐digit figures. However, in Japan, more favorable outcomes were reported by Doctor Nakayama, including 23 patients who survived for 5 years.^6^ This achievement became the focus of global attention.

The first meeting of the Japanese Society for Esophageal Diseases, hosted by Keio University's Professor Ichiro Akakura, focused on the postoperative complications of esophageal cancers and reconstruction methods. The rules for handling esophageal cancers were also examined, leading to the 1969 publication of the first edition of the Clinical Classification for Carcinoma of Esophagus.^8^ Since then, the safety of esophageal cancer surgery has improved, and long‐term outcomes have been considered an important issue.

In 1974, at the Annual Meeting hosted by Professor Kiyoshi Inokuchi (Kyushu University), the lymph node metastases of esophageal cancers were discussed. In a 1981 report by Professor Sannohe et al,^9^ from Fukuoka University, the possibility of metastasis to the cervical lymph nodes was recognized. When the Annual Meeting was hosted by Professor Tadayoshi Takemoto (Yamaguchi University) in 1985 and by Professor Takayoshi Tobe (Kyoto University) in 1986, “the actual condition of lymph node metastasis and the countermeasures thereof” and the “rational lymph node dissection and its extent” were selected as the main topics. These events led to discussions regarding cervical lymph node dissection and the importance of superior mediastinal lymph node dissection. In parallel, the relationship between the numbers of lymph node metastases and prognoses started to be discussed. When the meeting was hosted by Doctor Masakatsu Yamamoto in 1987, rational lymph node dissection was discussed again. At that time, bilateral cervical lymph node dissection had been performed at only six facilities, and the mortality rate among elderly patients was relatively high. Therefore, bilateral cervical lymph node dissection was not recognized as the standard treatment, but became an important discussion topic.

From then, the number of facilities performing three‐field lymph node dissection gradually increased. In 1990, when the meeting was hosted by Professor Kaichi Isono from the Second Department of Surgery at Chiba University School of Medicine, a nationwide questionnaire survey on two‐ and three‐field lymph node dissection was conducted. The results were published in the Journal of Oncology in 1991, showing that patients who underwent three‐field lymph node dissection had more favorable prognoses, with a five‐year survival rate of 34.3% compared with the 26.7% rate associated with two‐field lymphadenectomy.^10^ Since that time, three‐field lymphadenectomy has been recognized and widely used as the standard treatment for thoracic esophageal cancer. However, globally, three‐field lymphadenectomy was only performed at a limited number of facilities. Although the favorable outcomes were achieved and recognized in Japan, the procedure is not acknowledged as the standard surgical treatment in other parts of the world. Although the surgical procedure is safe, the surgical treatment of esophageal cancer is typically extremely invasive, deeming perioperative management critical for successful treatment outcomes. In the 1970s, central venous parenteral nutrition, high‐calorie infusions, and postoperative treatment using intermittent positive pressure breathing were introduced, and research studies examined the criteria for determining proper preoperative nutrition. Additionally, elemental diets started to be used for postoperative nutritional management. Moreover, Ando et al^11^ reported the usefulness of Swan‐Ganz catheters for circulatory management. The use of rapid turnover proteins as a nutritional index was also first studied in the 1980s. More recently, the use of immune‐nutrition, utilizing omega‐3 fatty acids for preoperative nutritional management, has been reported to be effective.^12^ Preoperative oral care was already covered by public health insurance in Japan, and was widely included in the treatment for all types of cancers; its effectiveness was also confirmed in esophageal cancer surgery patients in whom respiratory complications are important.

For the 2017 guidelines, a meta‐analysis of the effects of preoperative respiratory therapy or rehabilitation was conducted.^13^ The findings showed that such therapy led to a significant decrease in the postoperative risk of pneumonitis and respiratory complications. A meta‐analysis by Engelman and Maeyens showed that administering perioperative steroids significantly reduced the postoperative complications and did not increase the number of cases of anastomotic leakage, establishing the usefulness of perioperative steroid administration.^14^

As mentioned above, preoperative radiotherapy was adopted as an adjuvant therapy for improving long‐term outcomes following the introduction of the three‐stage surgical procedure. When the Society meeting was hosted by Doctor Toshio Mitomi in 1984, the main agenda was the evaluation of the long‐term outcomes of preoperative radiotherapy, its current indications, usefulness, and prognostic factors. Back then, favorable prognoses were already reported in cases showing the effectiveness of treatment. To further improve outcomes, postoperative adjuvant therapies were examined. In 1989, when the Society's meeting was hosted by Professor Takao Hattori (Hiroshima University), chemotherapy and immunotherapy were selected as the main topics, with adjuvant therapies being considered; the findings failed to show any clear usefulness for the adjuvant therapies. A clinical study, examining the usefulness of various post‐ and preoperative adjuvant therapies, was carried out by the Japanese Oncology Group (JCOG). In the JCOG 8806 study, postoperative adjuvant therapy using cisplatin (CDDP) and vindesine was compared with surgery alone; the findings showed no benefit associated with the adjuvant therapies. Later, in the JCOG9204 study, compared with the surgery‐alone group, a postoperative combination of CDDP and 5‐fluorouracil was shown to be useful against resectable esophageal cancers. Next, in the JCOG9907 study, preoperative chemotherapy was found useful,^15^ and is now recognized as the current standard treatment. Further, preoperative radiotherapy, in combination with chemotherapy, is recognized as a standard treatment, especially in the USA. To further improve patient prognoses, JCOG is currently conducting a multicenter randomized controlled study in Japan, to verify the utility of standard preoperative chemotherapy, preoperative chemoradiation therapy, and chemotherapy using three different drugs, including docetaxel; patient enrollment is complete, and the results are expected.

Presently, the surgical outcomes for esophageal cancer have stabilized and the procedure is safe. However, because the three‐field lymph node dissection (resection of cervical, mediastinal, and upper abdominal lymph nodes) is highly invasive, the development of less invasive surgical procedures has also progressed. Okazumi et al^16^ previously reported that even the ingenuity of the thoracotomy reduces the invasiveness of the surgery.

Specifically, postoperative systemic inflammatory response syndrome lasting 0.5 ± 0.7 days is possible when the latissimus dorsi and serratus anterior muscles are preserved through the use of a vertical skin incision; a muscle‐sparing thoracotomy is performed, without cutting the ribs and costal cartilage (with differential lung ventilation); reconstruction is carried out through the posterior mediastinal route, using a gastric tube on the side of the greater curvature of the stomach, preserving the omentum; and a mediastinal lymph node dissection is performed while preserving the thoracic duct.

Therefore, the endoscopic surgery has gained widespread application, with the first report by Cuschieri et al,^17^ in 1992 for surgical treatment of esophageal cancer. In Japan, endoscopic surgery for esophageal cancer was initially performed in 1995 by Akashi et al^18^ (Tohoku University). Since then, endoscopic surgery for the treatment of esophageal cancer gained widespread applicability. However, its long‐term outcomeshave not yet been demonstrated, and the procedure is only weakly recommended in the 2017 guidelines. Additionally, a safety analysis, conducted by Takeuchi et al^19^ in 2011, examined National Clinical Database (NCD) cases and showed that the incidence of complications was significantly higher and the number of repeat surgeries within 30 days was also significantly higher in the thoracoscopy group than in the open surgery group. Currently, JCOG is conducting a multicenter randomized controlled study; the results are expected in the near future.

The concept of robot‐assisted surgery, using the DaVinci surgical system, was first presented in 2004 by Bodner et al.^20^ Whereas, in Japan, Uyuma et al first reported its application in 2011. This surgical method has been approved for insurance coverage since 2018, and the number of cases is expected to increase.

Non‐open thoracic surgery, using a mediastinoscope, has also been a focus of attention. In 1933, non‐open thoracic surgery, involving access from the neck and the esophageal hiatus, was reported by Turner^21^; in Japan, it was first reported as a blunt dissection by Akiyama et al,^22^ in 1971. In a compilation of 35 cases, Buess also reported on the utility of surgeries using mediastinoscopes.^23^ In Japan, a report on surgeries using mediastinoscopes was published in 2004 by Tangoku et al^24^ from Yamaguchi University. In 2013, Mori et al^25^ (Tokyo University) reported cases of closed‐chest surgeries, involving access from the neck or esophageal hiatus, performed in combination with the DaVinci system. Since this system could possibly be applied in cases unsuitable for thoracotomy, future developments are expected.

In the Union for International Cancer Control (UICC) TNM classification, the supraclavicular lymph nodes are not classified as regional lymph nodes in thoracic esophageal cancer; instead, they are classified as M1 (LYM) distant metastases. Therefore, when a metastasis is found in the supraclavicular lymph nodes, thoracic esophageal cancer is classified as stage IV, according to the 7th (2009) edition of the classification, and stage IVB, according to the 8th (2017) edition. In Japan, the three‐field lymph node dissection has been recognized as the standard treatment. However, in the 11th (2015) edition of the Japanese Classification of Esophageal Cancer,^26^, ^27^ the results of an analysis of patients registered nationwide showed that supraclavicular lymph nodes were involved in the middle intrathoracic esophageal cancers classified as N2 and that bilateral cervical lymph node dissection was necessary in cases of D2 lymph node dissection. Prior to the 10th (2007) edition, these lymph nodes had been classified as the N3 group. The results of this analysis of nationwide cases were published, and one extremely interesting finding was that, in middle intrathoracic esophageal cancers, the rate of metastasis to the supraclavicular lymph nodes is higher than the rate of metastasis to the middle mediastinum lymph nodes.^28^ Conversely, the 5‐year survival rate of patients with supra‐clavicular node metastases is higher than that of patients with middle‐mediastinal node metastases. Based on those findings, supraclavicular lymph nodes were reclassified into the N2 group in the 11th edition. In addition, according to the 7th edition of the TNM classification of resected esophageal cancers, registered in 2011, the 5‐year survival rate for pStage IV disease was 22.8%, but was 14.8% for pStage IIIc disease, showing inverted tendencies.^29^ There is a need to universally recognize supraclavicular lymph node metastases as local lymph node metastases and not as distant metastases.

Regardless of the progress made to date, unresectable esophageal cancers and recurrent esophageal cancers remain difficult to treat; therefore, additional new treatments need to be developed.

## 4 CONCLUSIONS

During the past 50 years, the surgical treatment of esophageal cancers has undergone significant progress, thanks to our predecessors' efforts, resulting in improved safety and prognoses compared with earlier techniques. Further, advances in radiation therapy and chemotherapy occurred in addition to recognizing the importance of multidisciplinary treatments and treatment options tailored to individual patients. In the future, more outcome improvements are expected, including in immunotherapy using immune checkpoint inhibitors—a class of therapies that has recently attracted attention as the fourth pillar of cancer therapy.

## DISCLOSURE

Conflict of Interest: The author declares no Conflict of Interests for this article.

## References

[1] Seo S . Esophageal surgery. Nippon Geka Gakkai Zasshi (J Jpn Surg Soc). 1933;33:1461–505 (in Japanese).

[2] Osawa T . Esophageal surgery. Nippon Geka Gakkai Zasshi (J Jpn Surg Soc). 1933;34:1319–590 (in Japanese).

[3] Torek F . The first successful case of resection of the thoracicportion of the oesophagus for carcinoma. Surg Gynecol Obstet. 1913;16:614–7.

[4] Kirschner M . Ein neues Verfahren der Oesophagoplastik. Arch Klin Chir. 1920;114:606–63.

[5] Ohsawa T . Ueber die ventro‐arco‐diphragmale Thorakolaparotomie bzw. Laparothorakotomie. Zentralbl Chir. 1930;40:2467–72.

[6] Nakayama K . Esophageal cancer – recent progress of surgical therapy for cancer. Jpn J Clin Exp Med. 1964;41:2035–41 (in Japanese).

[7] Nakayama K . Experiences of about 3,000 cases with cancer of the esophagus and the cardia. Aust N Z J Surg. 1962;31:222–30. 14478237 10.1111/j.1445-2197.1962.tb03266.x

[8] The Japanese Society for Esophageal Diseases . Clinical classification for carcinoma of esophagus. Tokyo, Japan: The Japanese Society for Esophageal Diseases; 1969.

[9] Sannohe Y , Hiratsuka R , Doki K . Lymph node metastases in cancer of the thoracic esophagus. Am J Surg. 1981;141:216–8. 7457740 10.1016/0002-9610(81)90160-4

[10] Isono K , Sato H , Nakayama K . Results of a nationwide study on the three‐field lymph node dissection of esophageal cancer. Oncology. 1991;48:411–20. 1745490 10.1159/000226971

[11] Ando N , Ohtaka H , Miyoshi H , Fujisaki M , Fukuda T , Abe O . Studies on pre‐ and postoperative extravascular lung water changes in patients with esophageal cancer. Nippon Geka Gakkai Zasshi (J Jpn Surg Soc). 1983;84:310–20. 6371485

[12] Daly JM , Weintraub FN , Shou J , Rosato EF , Lucia M . Enteral nutrition during multimodality therapy in upper gastrointestinal cancer patients. Ann Surg. 1995;221:327–38. 7726669 10.1097/00000658-199504000-00002 PMC1234581

[13] Kitagawa Y , Uno T , Oyama T , Kato K , Kato H , Kawakubo H , et al. Esophageal cancer practice guidelines 2017 edited by the Japan esophageal society: part 2. Esophagus. 2019;16:25–43. 30171414 10.1007/s10388-018-0642-8 PMC6510875

[14] Engelman E , Maeyens C . Effect of preoperative single‐dose corticosteroid administration on postoperative morbidity following esophagectomy. J Gastrointest Surg. 2010;14:788–804. 20229072 10.1007/s11605-010-1168-0

[15] Ando N , Kato H , Igaki H , Shinoda M , Ozawa S , Shimizu H , et al. A randomized trial comparing postoperative adjuvant chemotherapy with cisplatin and 5‐fluorouracil versus preoperative chemotherapy for localized advanced squamous cell carcinoma of the thoracic esophagus (JCOG9907). Ann Surg Oncol. 2012;19:68–74. 21879261 10.1245/s10434-011-2049-9

[16] Okazumi S , Ochiai T , Shimada H , Matsubara H , Nabeya Y , Miyazawa Y , et al. Development of less invasive surgical procedures for thoracic esophageal cancer. Dis Esophagus. 2004;17:159–63. 15230731 10.1111/j.1442-2050.2004.00379.x

[17] Cuschieri A , Shimi S , Banting S . Endoscopic oesophagectomy through a right thoracoscopic approach. J R Coll Surg Edinb. 1992;37:7–11. 1573620

[18] Akaishi T , Kaneda I , Higuchi N , Kuriya Y , Kuramoto J , Toyoda T , et al. Thoracoscopic en bloc total esophagectomy with radical mediastinal lymphadenectomy. J Thorac Cardiovasc Surg. 1996;112:1533–41. 8975845 10.1016/s0022-5223(96)70012-0

[19] Takeuchi H , Miyata H , Ozawa S , Udagawa H , Osugi H , Matsubara H , et al. Comparison of short‐term outcomes between open and minimally invasive esophagectomy for esophageal cancer using a nationwide database in Japan. Ann Surg Oncol. 2017;24:1821–7. 28224366 10.1245/s10434-017-5808-4

[20] Bodner J , Wykypiel H , Wetscher G , Schmid T . First experiences with the da Vinci operating robot in thoracic surgery. Eur J Cardiothorac Surg. 2004;25:844–51. 15082292 10.1016/j.ejcts.2004.02.001

[21] Tuner G . Excision of thoracic esophagus for carcinoma with construction of an extra‐thoracic gullet. Lancet. 1933;2:1315–6.

[22] Akiyama H , Sato Y , Takashashi F . Immediate pharyngogastrostomy following total esophagectomy by blunt dissection. Jpn J Surg. 1971;1:225–31. 5173077 10.1007/BF02468912

[23] Buess G . Thoracoscopic dissection of the esophagus. Surg Endosc. 1992;6:150–1. 1502683 10.1007/BF02309091

[24] Tangoku A , Yoshino S , Abe T , Hayashi H , Satou T , Ueno T , et al. Mediastinoscope‐assisted transhiatal esophagectomy for esophageal cancer. Surg Endosc. 2004;18:383–9. 14735343 10.1007/s00464-003-8181-2

[25] Mori K , Yamagata Y , Wada I , Shimizu N , Nomura S , Seto Y . Robotic‐assisted totally transhiatal lymphadenectomy in the middle mediastinum for esophageal cancer. J Robot Surg. 2013;7:385–7. 24273609 10.1007/s11701-013-0398-z PMC3835922

[26] Japan Esophageal Society . Japanese Classification of Esophageal Cancer, 11th Edition: part I. Esophagus. 2017;14:1–36. 28111535 10.1007/s10388-016-0551-7 PMC5222932

[27] Japan Esophageal Society . Japanese Classification of Esophageal Cancer, 11th Edition: part II and III. Esophagus. 2017;14:37–65. 28111536 10.1007/s10388-016-0556-2 PMC5222925

[28] Tachimori Y , Ozawa S , Numasaki H , Matsubara H , Shinoda M , Toh Y , et al. Efficacy of lymph node dissection by node zones according to tumor location for esophageal squamous cell carcinoma. Esophagus. 2016;13:1–7. 26752982 10.1007/s10388-015-0515-3 PMC4698372

[29] Tachimori Y , Ozawa S , Numasaki H , Ishihara R , Matsubara H , Muro K , et al. Comprehensive Registry of Esophageal Cancer in Japan, 2011. Esophagus. 2018;15:127–52. 29948477 10.1007/s10388-018-0614-z PMC6021481

